# Ciliary Videomicroscopy: A Long Beat from the European Respiratory Society Guidelines to the Recognition as a Confirmatory Test for Primary Ciliary Dyskinesia

**DOI:** 10.3390/diagnostics11091700

**Published:** 2021-09-17

**Authors:** Noemie Bricmont, Mihaela Alexandru, Bruno Louis, Jean-François Papon, Céline Kempeneers

**Affiliations:** 1 Pneumology Laboratory, I3 Group, GIGA Research Center, University of Liège, 4000 Liège, Belgium; ckempeneers@chuliege.be; 2 ENT Department, Assistance Publique-Hôpitaux de Paris (AP-HP), Université Paris-Saclay, Hôpital Bicêtre, 94270 Le Kremlin-Bicêtre, France; mihaeladana.alexandru@aphp.fr (M.A.); jean-francois.papon@aphp.fr (J.-F.P.); 3 Institut Mondor de Recherche Biomédicale INSERM-UPEC UMR 955, CNRS ERL7000, 94010 Créteil, France; bruno.louis@inserm.fr; 4 Division of Respirology, Department of Pediatrics, University Hospital Liège, 4000 Liège, Belgium

**Keywords:** primary ciliary dyskinesia (PCD), digital high-speed videomicroscopy (DHSV), ciliary beat frequency (CBF), ciliary beat pattern (CBP), diagnostic, standardization

## Abstract

Primary ciliary dyskinesia (PCD) is a rare inherited ciliopathy in which respiratory cilia are stationary or dyskinetic. The clinical presentation of PCD is highly non-specific since it includes infections and disorders of the upper (otitis and rhinosinusitis) and lower (neonatal respiratory distress, bronchitis, pneumonia and bronchiectasis) airways, starting in early life. Clinical examination alone does not allow a PCD diagnosis, which relies on several concordant tests, since none are sensitive or specific enough alone. Despite being the most sensitive and specific test to diagnose PCD, digital high-speed videomicroscopy (DHSV) is not sufficiently standardized, preventing its use with complete confidence as a confirmatory diagnostic test for PCD, or its inclusion in a diagnostic algorithm. Since the 2017 ERS recommendations for PCD diagnosis, three main issues remain to be solved in order to optimize DHSV ciliary beating evaluation: the problem in defining an accurate sensitivity and specificity as there is no gold standard method to diagnose all PCD cases, a lack of standardization in the operating procedure for processing respiratory samples, and in the choice of measured parameters (self-operating or not). The development of new automated analysis approaches is promising and will require full clinical validation.

## 1. Introduction

Mucociliary transport is a key innate mechanism of airway defense. It relies upon the continuous periodic and coordinated beating of respiratory motile cilia that removes the pathogens trapped in the mucus layer. In case of beating disorders, the inefficiency of mucociliary transport leads to pathogen stagnation, proliferation and airway infections.

Primary ciliary dyskinesia (PCD) is a group of inherited diseases characterized by mucociliary transport defects due to inefficient beating. PCD is a rare disease, affecting 1 in every 10,000–40,000 individuals born [1]. The clinical presentation of PCD is highly non-specific since it includes infections and disorders of the upper (otitis and rhinosinusitis) and lower (neonatal respiratory distress, bronchitis, pneumonia and bronchiectasis) airways, starting in early life. However, some syndromic associations point to PCD. Notably, nearly 50% of PCD patients present with situs inversus secondary to abnormal beating of the primary nodal motile cilia, responsible for organ lateralization [2]. The association of rhinosinusitis, bronchiectasis and situs inversus constitutes the well-known Kartagener syndrome which is considered as a clinically diagnosed PCD [3]. The phenotype can also include congenital cardiac malformations, laterality defects other than situs inversus and fertility issues secondary to an abnormal beating of cilia in the female reproductive tract or to an immotile flagellum (a cilium-like structure) of the male spermatozoid [4].

Clinical examination alone does not allow a PCD diagnosis, which relies on several concordant tests, since none are sensitive or specific enough alone. PCD diagnostic testing includes ciliary beating observation, ciliary ultrastructure study by transmission electron microscopy (TEM), immunostaining of ciliary proteins, nasal nitric oxide measurement and molecular sequencing [5]. In clinical practice, analyses of ciliary beating are not feasible in vivo and require sampling respiratory ciliated cells from the nose or bronchus for ex vivo study [6,7,8]. In selected cases, epithelial cells can be cultured for in vitro analysis to differentiate PCD from secondary dyskinesia [9,10,11].

Microscopic visualization of immotile respiratory cilia was one of the first observations of abnormal ciliary motion developed for PCD diagnosis, reported as “immotile cilia syndrome” at that time [12,13]. Later, the observation of motile but dyskinetic cilia in a case of Kartagener syndrome led to the name of PCD [14]. Meanwhile, coupling the microscope with stroboscopy, photo-oscillometer or photo-multiplier techniques allowed measuring the ciliary beat frequency (CBF), whose ex vivo normal values are between 10 and 15 Hz at 37 °C [15]. For several years, decreased CBF was a criterion for performing a TEM, which was the gold reference test for diagnosis [16]. However, some PCD cases present with a normal CBF, while a low CBF can also be secondary to airborne aggressions (tobacco smoke, pollution) and to non-PCD respiratory diseases such as rhinosinusitis and chronic bronchitis [17,18,19,20,21]. Thus, the limits of the early techniques led to the development of digital high-speed videomicroscopy (DHSV). This technique uses a high-speed digital video camera attached to a microscope that records ciliary beating at high speeds (120 to 500 frames per second) [15].

Proposed for ciliary studies since 1984 [22], DHSV has resulted in a major improvement in ciliary beating analysis, allowing multiple slow-motion replays and providing video collections suitable for expert advice or research purposes [5,15,23].

Using DHSV, Chilvers et al. suggested that the ciliary beat pattern (CBP) could be associated with specific ultrastructural defects [23]. Moreover, Stannard et al. demonstrated a superior accuracy of CBP evaluation using DHSV compared with CBF measurement alone [16]. To be able to evaluate CBP precisely, a video playback system allows the video sequences to be reviewed at a reduced frame rate, and observing the precise movement of cilia during their beat cycles is necessary [24]. Moreover, the quality of samples is important. Indeed, abundant ciliated cell samples and undisrupted ciliated epithelial edges at least 50 µm in length and free of mucus, germs and cellular debris are important to characterize CBP [24], as Thomas et al. [25] showed that a disrupted ciliated epithelium showed a slower CBF and increased dyskinesia.

Later, Papon et al. demonstrated that DHSV allowed a detailed analysis of ciliary beating not only by subjectively describing the ciliary beat patterns (i.e., normal, virtually immotile, stiff beating with a reduced amplitude, circular gyrating motion) but also by measuring numerous objective parameters (e.g., CBF, power and recovery stroke duration, pauses after the active and recovery strokes, cilia length and beating angle, distance traveled by the cilium and the area swept) [26]. To date, several studies have correlated ciliary beating anomalies with particular ultrastructural defects and genotypes [27,28].

Considering the importance of DHSV for PCD diagnosis, the European Respiratory Society (ERS) Task Force on PCD diagnosis stated in 2017 that DHSV is an accurate test for PCD when performed by experienced observers combining CBF measurement and CBP analysis (sensitivity of 0.95–1.00, and specificity of 0.93–0.95) [5]. The guidelines were as follows:

- High-speed video analysis, including CBF and CBP analysis, should be used as part of the diagnostic work-up of patients suspected of having PCD (weak recommendation);
- CBF should not be used without assessment of CBP in diagnosing PCD (strong recommendation);
- To improve the diagnostic accuracy of DHSV, CBF and CBP assessment should be repeated after air–liquid interface epithelial cell culture (strong recommendation) [5].

While PCD diagnosis is obvious when the clinical suspicion is high and cilia are immotile, the 2017 ERS guidelines highlighted the limits of DHSV, which is not sufficiently standardized to rule in or rule out PCD in isolation. Since then, new guidelines and research development have attempted to improve objective and standardized ciliary beating analysis.

## 2. Update of Literature Concerning Ciliary Videomicroscopy since the 2017 European Respiratory Society Recommendation, Concerning Ciliary Videomicroscopy Sensitivity and Specificity for PCD, and Advances in the Standardization of the Technique

The ERS recommendations on DHSV as a PCD diagnostic test were based on low confidence due to three main issues: the lack of evidence on DHSV’s diagnostic efficiency (1), the lack of consensus on a standard operating procedure to perform DHSV (2), and on the measured parameters for ciliary beating evaluation (3).

### 2.1. The Lack of Evidence on DHSV’s Diagnostic Efficiency

Some new studies focused on the evaluation of DHSV’s reliability for PCD diagnosis, but only two were retained in the ERS guidelines to evaluate the sensibility and specificity of DHSV [5], as these were selected according to the GRADE approach [29]. However, this might be an inaccurate evaluation of DHSV’s diagnostic reliability as it was evaluated against an incomplete reference standard (mainly TEM alone) or against a reference standard including DHSV. A recent retrospective cohort study published in 2019 by Rubbo et al. reported an excellent sensitivity (100%) and specificity (96%) for DHSV to diagnose PCD against the ERS guidelines (TEM and/or genetic testing) [30], but this retrospective study was criticized for its methodology [31]. Indeed, Shapiro et al. [31] highlighted that in this study, the scientists selected six video clips per patient, rather than randomly selecting video clips from an eligible pool, suggesting a possible inappropriate exclusion of clips. Furthermore, they raised the issue that DHSV was not performed on three occasions or after cell culture, as recommended by the ERS guidelines, suggesting that secondary dyskinesia might be wrongly interpreted as a PCD diagnosis. However, DHSV is highly important in PCD diagnosis as it might detect PCD cases missed by TEM and/or genetics. The 2017 ERS guidelines recognized that TEM and genetics together would miss 20–30% [5] of patients who truly have PCD (false negative), and DHSV might detect most of these patients who require specialist PCD care [30]. For instance, in patients with a mutation in the PCD-associated genes *DRC2*, *OFD1*, *GAS2L2*, *LRRC56*, *CFAP57*, *CFAP221*, *SPEF2* and *DNAH11*, ciliary ultrastructural evaluation by TEM is mainly normal [28]. Before the discovery of these genes, PCD diagnosis was highly suggested only by abnormal ciliary beating detected by DHSV [32].

### 2.2. The Lack of Consensus on a Standard Operating Procedure to Perform DHSV

The second issue is that the DHSV protocol is not sufficiently standardized to confirm a PCD diagnosis [5,33]. Indeed, the respiratory epithelium can be sampled using a brush, a curette or forceps, usually from the nose [34]. Moreover, ciliary function varies under differing environmental and physiological conditions, such as pH [35,36,37], osmolarity [36,37], oxygen [38], the presence of drugs [39], vitamins [40] or ions [41] within the medium, the temperature [35,42] (with some centers measuring at 37 °C [26,43,44,45] and others at lower temperatures [7,9,46]) and the time between sampling and video recording [47]. These conditions need to be strictly regulated, since minor variations may affect ciliary function.

Since the 2017 ERS recommendations, only three standard operating procedure studies have been published.

Nikolaizik et al. [48] studied CBF and CBP using DHSV (with a Sisson-Ammons Video Analysis) at three different temperatures: 25 °C, 32 °C and 37 °C, on 100 young healthy volunteers. As with previous studies, the results showed that the CBF increases significantly with the temperature. Indeed, the median CBF was 7.0 Hz (6.2–9.6), 7.6 Hz (5.8–9.1) and 8.5 Hz (6.5–9.8) at 25 °C, 32 °C and 37 °C, respectively (*p* < 0.0001). They also reported that the CBP did not change according to the temperature. Unfortunately, they did not report a precise description of the beating pattern, and no quantitative CBP evaluation was available.

Bricmont et al. [44] studied the influence of the conservation of respiratory epithelial samples on ciliary functional analysis to determine the optimal storage conservation and durations of nasal brushing samples before being prepared for DHSV. In this study, using only five nasal brushing samples from healthy adults, samples were divided equally and conserved either at 4 °C or at 22 °C. Beating cilia were recorded using DHSV at 37 °C immediately (H0), and then 9 h after sampling (H9). Ciliary function was assessed by the CBF and the percentage of normal CBPs. The results showed that there was no significant difference between the CBF assessed immediately or 9 h after sampling, regardless of the sample storage temperature (13.4 ± 1.9 Hz (H0) vs. 14.9 ± 2.8 Hz (H9 at 4 °C), *p* = 0.44, and 13.4 ± 1.9 Hz (H0) vs. 17.1 ± 3.1 Hz (H9 at 22 °C), *p* = 0.11). Similar results have been shown for the percentage of normal CBPs: 80 ± 7.5% (H0) vs. 70.3 ± 8.5% (H9 at 4 °C) (*p* = 0.16), and 80 ± 7.5% (H0) vs. 78.5 ± 8.1% (H9 at 22 °C) (*p* = 0.70) [44].

Reula et al. [49] studied ciliary motility variations with time and temperature. In this study, 27 nasal curettages from healthy volunteers were divided equally; half of the samples were kept at room temperature (22–24 °C), and the other half were kept in a refrigerator (4 °C). For each sample, video sequences were recorded using DHSV at room temperature at 0, 3, 24 and 48 h after sampling. Three samples were also recorded at 72 h. The results showed that both at room temperature and at 4 °C, the CBF increased with the time between sampling and video recording. The percentage of dyskinetic CBPs also increased with the time before video recording, especially after 3 h, and regardless of the storage temperature, suggesting that video recording should be performed within 3 h after sampling. However, in 2010, Sommer et al. [47] reported a moderate increase in the CBF (recorded at 22 °C) during the first 3 h, followed by a slow decrease, with the greatest stability between 3 and 9 h after sampling; no description of the CBP was reported.

Unfortunately, these three studies were performed only on healthy volunteers.

Lee et al. [50] tested four different commercially available cell culture media (BEGM (Bronchial Epithelial Cell Growth Medium, Lonza), AECGM (Airway Epithelial Cell Growth Medium, PromoCell), LHC-8 (Gibco) and PneumaCult (STEMCELL Technologies)) for respiratory ciliated epithelial cell differentiation of nine nasal brushing samples from six healthy subjects, two patients with chronic obstructive pulmonary disease and one patient with PCD, using the ALI cell culture method. During the ALI cell culture procedure, the ciliated respiratory epithelium is in contact with the cell culture medium for a long duration (between 21 and 42 days [51]). They reported that the CBF was influenced by the cell culture medium chosen. Indeed, cilia obtained from the ALI culture using PneumaCult had a statistically lower CBF than those from AECGM (*p* < 0.01) or LHC-8 (*p* < 0.001) [50]. These results suggested that a long-term exposure to varying compositions and concentrations of nutrients present in the culture medium may influence ciliary beating. However, no evaluation of the CBP was performed.

These four standard operating procedure studies bring some new insights on the DHSV methodology, but larger studies are needed to confirm these preliminary results, notably in pathological conditions.

Given the lack of evidence-based data and formal recommendations for the optimal respiratory sample collection and processing, various laboratories use different protocols and consequently have different reference values [52].

### 2.3. The Lack of Consensus on the Measured Parameters for Ciliary Beating Evaluation

The third issue highlighted regarding DHSV is that the choice of the parameters set to analyze ciliary beating is not clearly established. Do we have to use a physical parameter defined in a specific region of interest (ROI) vs. a whole field analysis (e.g., CBF or amplitude)? How can we obtain the mean of this parameter to obtain a representative value of the studied subject? Do we have to use a global score (e.g., CBP, shear stress, coordination) or an association of parameters? These remain open-ended questions.

Despite these criticisms, this technique has many considerable advantages. Indeed, DHSV provides an accurate result on the day of the testing, which allows direct patient counselling and direct appropriate care while awaiting confirmatory TEM and genetics [30]. DHSV may also have another important advantage in the assessment of new PCD treatments. Indeed, as the whole procedure is relatively easy to repeat over time, it might help to assess the treatment’s ability to restore or degrade ciliary function, and to longitudinally follow up the ciliary function in PCD patients.

## 3. New Applications of Videomicroscopy, Focusing on New Approaches for Automated Ciliary Motion Evaluation

As manual processing of DHSV data involves some subjectivity and is time-consuming [53,54], a variety of software applications have been developed for CBF and CBP assessment, using different semi-automated [54,55] (involving the selection of specific ROIs) or fully automated programs [53,56] (analyzing the entire captured image). Different computer-assisted software packages for CBP analysis [57] have been developed, mostly involving the evaluation of the CBP in a limited ROI, but currently, none are commercially available. Based upon DHSV, several methods have recently been proposed to evaluate, both quantitatively and almost automatically, the ciliary motion and/or its efficiency.

In 2015, Quinn et al. [58] proposed using the spatial and temporal variations in the optical flow to define a digital signature characterizing the ciliary motion. Briefly, optical flow models the apparent motion at each pixel from frame to frame. This motion is decomposed into two elemental components of rotation and deformation. Then, the frequency histograms of these components define the digital signature of the ciliary motion. The authors found that this digital signature allowed differentiating normal and abnormal ciliary motions with a high degree of accuracy.

Bottier et al. [59,60] proposed assessing the cilia movement by tracking the movement of microbeads used as markers of the fluid displacement generated by ciliary beating. For this, 4.5 µm-diameter microbeads are inserted in the cell suspension, and their motion next to the ciliated edge is recorded by DHSV. The microbead motion provides the fluid velocity field, which allows estimating the shear stress induced by the cilia on the fluid if we know the fluid viscosity. This shear stress characterizes the momentum transfer between the cilia and fluid. It can be seen as an index of the ciliary motion efficiency.

Feriani et al. [61] proposed using the differential dynamic microscopy-based approach [62] to perform, simultaneously and in a completely automated fashion, both a temporal and a spatial analysis on top-view DHSV of a layer, which allows estimating the CBF and the degree of cilia coordination. Here, the degree of cilia coordination is not the wavelength of the metachronal wave but a scale where the cilia dynamics is coordinated. The method based upon the frame differences at various times and the 2D Fourier transform may provide temporal and spatial coherence of any dynamics in the video. In the context of cystic fibrosis, Chioccioli et al. [63] observed that the coordination in air–liquid interface epithelial cell cultures was affected by CFTR-modulating drugs.

If these three methods clearly provide global information about ciliary motion with either the motion signature, its coordination or its effectiveness without requiring too much subjectivity of an operator, it nevertheless remains true that these methods have not been systematically confronted with cohorts of PCD. Thus, to date, there has been no study in the literature that allows describing the accuracy of these methods in the diagnosis of PCD and/or their ability to differentiate different genotypes/phenotypes encountered in PCD.

CBP manual evaluation and characterization of specific beating patterns are currently highly subjective; however, automated methods currently do not allow defining a particular CBP. Further research has to focus on developing automated beat pattern recognition. This will require a high number of video sequences of ciliary beating obtained from well-defined PCD phenotypes, in order to allow correlations between specific CBPs and specific ciliary ultrastructural and genetic defects. As PCD is a rare disease, this will require collaboration between international PCD centers, using a standardized operating procedure to perform DHSV.

## 4. Conclusions

Despite being the most sensitive and specific test to diagnose PCD, international recommendations have stated that DHSV is not sufficiently standardized to be used as a confirmatory diagnostic test (European Respiratory Society) [5], or to be included in a diagnostic algorithm (American Thoracic Society) [33].

Since the 2017 ERS recommendations for PCD diagnosis, three main issues persist, preventing DHSV from being used as a confirmatory diagnostic test for PCD: the difficulty in defining an accurate sensitivity and specificity for DHSV as there is no gold standard test detecting all PCD cases, a lack of standardization in the operating procedure for processing samples using DHSV, and in the interpretation of ciliary functional analysis.

Rigorous quantitative methodological studies are needed to study the impact of different conditions used in the operating procedure for DHSV on complete ciliary function. Once this is identified, an expert consensus has to define “standard conditions” to perform DHSV, and a standardized CBF and CBP evaluation system. Using this standardized operating procedure and ciliary functional evaluation, normal data can be defined and used in different laboratories for a consensual PCD diagnosis. Moreover, as a manual evaluation can be performed only by experienced scientists, the standardization of the manual DHSV operation is the first step to developing a standardized automated ciliary beating evaluation, especially to be used in the clinical field and in new PCD diagnostic centers.
